# Transcriptome Analysis Reveals the Potential Molecular Mechanisms of Tiller Bud Development in Orchardgrass

**DOI:** 10.3390/ijms242115762

**Published:** 2023-10-30

**Authors:** Xiaoheng Xu, Guangyan Feng, Zhongfu Yang, Qiuxu Liu, Gang Nie, Dandan Li, Ting Huang, Linkai Huang, Xinquan Zhang

**Affiliations:** College of Grassland Science and Technology, Sichuan Agricultural University, Chengdu 611130, China

**Keywords:** strigolactone, sucrose, orchardgrass, tiller bud, development

## Abstract

Tillering is a special type of branching and one of the important contributors to the yield of cereal crops. Strigolactone and sucrose play a vital role in controlling tiller formation, but their mechanism has not been elucidated completely in most crops. Orchardgrass (*Dactylis glomerata* L.) is an important perennial forage with prominent tillering ability among crops. To date, the mechanism of tillering in orchardgrass is still largely unknown. Therefore, we performed a transcriptome and miRNA analysis to reveal the potential RNA mechanism of tiller formation under strigolactone and sucrose treatment in orchardgrass. Our results found that *D3*, *COL5*, *NCED1*, *HXK7*, miRNA4393-z, and miRNA531-z could be key factors to control tiller bud development in orchardgrass. In addition, strigolactones might affect the ABA biosynthesis pathway to regulate the tiller bud development of orchardgrass, which may be related to the expression changes in miRNA4393-z, *NCED1*, and *D10*. miRNA531-z could be involved in the interaction of strigolactones and sucrose in regulating tillering. These results will be further used to clarify the potential mechanism of tillering for breeding new high-tillering and high-production orchardgrass varieties and beneficial to improving the production and reproduction of crops.

## 1. Introduction

Shoot branching is one of the main drivers in the establishment of plant architecture and is considered an important economic trait for agriculture [1]. Tillering is a special type of branching and one of the important contributors to the yield of cereal crops. It is mainly composed of two processes—tiller bud formation and elongation. LAX2 acts together with LAX1 in rice to control tiller formation by regulating the process of axillary meristem (AM) formation [2,3]. MOC1 functions as a co-activator of MOC3 to activate *FON1* and control tiller bud outgrowth in rice [4]. Moreover, gibberellins (GAs) inhibit SLR1 expression by binding to GID1 to control tiller shoot formation [5]. Strigolactones (SLs) mainly regulate tiller bud elongation by promoting the degradation of the D53 protein, a repressor of strigolactone signaling in rice [6]. SLs induce D53 degradation in a DWARF14 (D14) and DWARF3 (D3)-dependent manner. D27 is involved in the biosynthesis of strigolactones, which regulate the outgrowth of tiller buds in rice [7]. In addition, the complex interactions between SLs and other phytohormones, such as ABA, IAA, and CKs, also play an important role in the regulation of tillering [8,9,10]. Among these, GA and ABA can promote tiller formation by inhibiting SL biosynthesis [10,11]. Cytokinins (CKs) and brassinosteroids (BRs) accelerate the development of tiler buds [12]. In addition, low phosphorus and low nitrogen inhibit tillering by inducing SL biosynthesis [13,14,15]. Nitrogen has a particularly substantial regulatory effect on the development of tiller buds [16]. A recent study reported that sucrose antagonizes the suppressive action of SLs on tillering by affecting the degradation of D53, a key component of SL signaling in rice [17]. Collectively, SLs play a central role in the regulation of plant architecture via multiple phytohormones and nutrients, and sucrose inhibits SL response by affecting the key components of SL signaling. However, the nuclear mechanisms of sucrose and SLs that coordinately control tiller formation are still unknown.

miRNAs are a type of approximately 21 nucleotide non-coding RNAs, and they bind to target mRNAs to repress their expression via cleavage and (or) translation repression at the post-transcriptional level in both plants and animals [18,19]. The overexpression of miR156 leads to tillering inhibition through the decreased expression of *IPA1* and *TB1* [20,21]. miR171c-targeted SCL6-II, SCL6-III, and SCL6-IV are important to regulate shoot branching in *Arabidopsis* [22]. miR160 fine-tunes auxin signaling to form more tillers by decreasing the accumulation of ARFs in rice [23,24]. OsmiR393 overexpression leads to more tillers via *OsTIR1* and *OsAFB2* downregulation in rice [25]. To date, a few miRNAs have been reported to participate in tillering regulation, but the function of miRNAs in regulating tiller formation remains largely unknown, especially in forage.

Orchardgrass (*Dactylis glomerata* L.) is an important perennial forage with great economic and ecological value native to temperate Asia, northern Africa, and western and central Europe [26]. Orchardgrass plays an important role in the supply of grass-fed meat, eggs, and milk throughout temperate regions owing to its prominent tillering ability, high nutritive value, adaptability, and biomass [27,28]. The regeneration capacity, nutrient yield, and seed yield of perennial forage are all partially or fully dependent on tillering. Therefore, tillering is vital to the yield and reproduction of forage and other crops. However, the mechanism of tillering has mainly focused on annual crops, such as rice, wheat, and so on, and the mechanism of tillering in perennial forage, such as orchardgrass, is still largely unknown. Orchardgrass is a good choice to study the molecular mechanism of tillering in perennial forage because of its outstanding tillering ability. Our previous study found that SLs can affect the tiller number in orchardgrass [29]. However, the role of sucrose as a new tiller regulator and SLs in the regulation of tiller bud development in orchardgrass is yet to be studied. Based on that, we performed a transcriptome and miRNA analysis to reveal the mechanism of tiller formation in orchardgrass by applying exogenous sucrose, SL, or both, and our results will be beneficial to improving the production and reproduction of forage and other crops.

## 2. Results

### 2.1. Morphology of Tiller Buds under Three Exogenous Treatments

**Figure 1:**
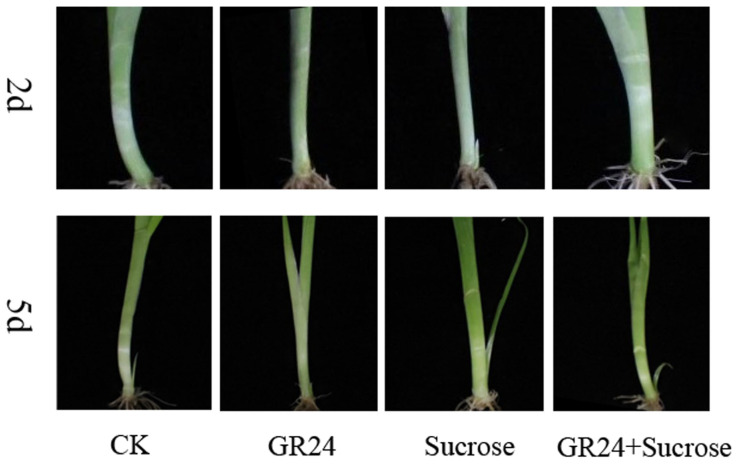
Morphology of tiller bud in orchardgrass 2 or 5 days after different exogenous treatments. CK represents no treatment; GR24 represents exogenous SL treatments; sucrose represents exogenous sucrose treatments; GR24+Sucrose represents exogenous SL and sucrose treatments.

Visible tiller buds were formed after 2 days of 4% sucrose treatment, while visible tiller buds did not form in the other treatment and control group (Figure 1). However, visible tiller buds were formed in all four groups after 5 days of treatment in each group (Figure 1). The smallest tiller buds were in the GR24 (an SL analog) treatment group and the longest tiller buds were in the sucrose treatment group. In addition, tiller buds were medium and developed similarly in control and GR24+sucrsose treatment groups (Figure 1). These results indicated that exogenous sucrose could promote the development of tiller buds, and exogenous SL can inhibit the development of tiller buds. When SL and sucrose were both applied, sucrose still slightly promoted tiller bud development, but the difference was not significant compared with the control group.

### 2.2. DEGs in Three Exogenous Treatment Groups

**Figure 2:**
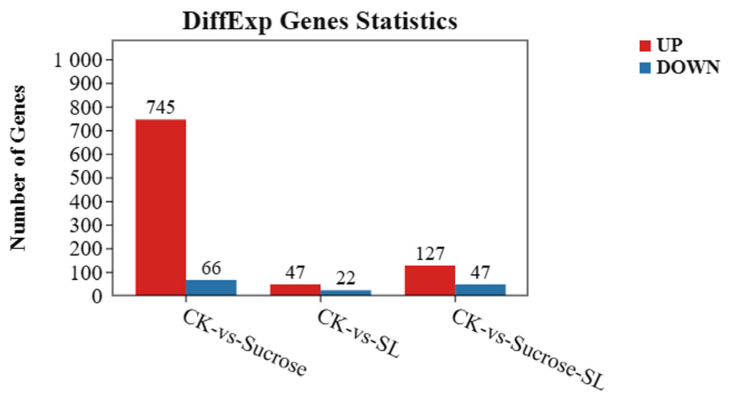
The number of differential-expression genes in three groups. The red column represents the number of upregulated genes, and the blue column represents the number of downregulated genes.

To reveal the molecular mechanism of phenotypic change caused by sucrose or SL, we performed a transcriptome and miRNA analysis to reveal the mechanism of tiller formation in orchardgrass by applying the exogenous sucrose, SL, or both. In transcriptome data, 811 significantly differentially expressed genes (745 upregulated and 66 downregulated) were found in the CK vs. Sucrose group (Table S3). There were 47 upregulated and 22 downregulated DEGs in the CK vs. SL group (Table S4), and 127 upregulated and 47 downregulated DEGs in the CK vs. SL+Sucrose group (Figure 2 and Table S5). Notably, the number of upregulated DEGs is more than the downregulated DEGs in each group, and the maximum number of upregulated DEGs is in the CK vs. Sucrose group. In particular, the number of DEGs in the CK vs. SL+Sucrose group is between the CK vs. Sucrose group and CK vs. SL group, and some DEGs in the CK vs. The SL+Sucrose group can respond to SL and sucrose. Combined with phenotypic changes in tiller buds under three exogenous treatments, these results indicated that SL and sucrose could be antagonistic to each other. SL plays a negative role, and sucrose plays a positive role in affecting the development of tiller buds.

### 2.3. GO and KEGG Pathway Analysis of CK vs. SL Group and CK vs. SL+SucroseL Group

**Figure 3:**
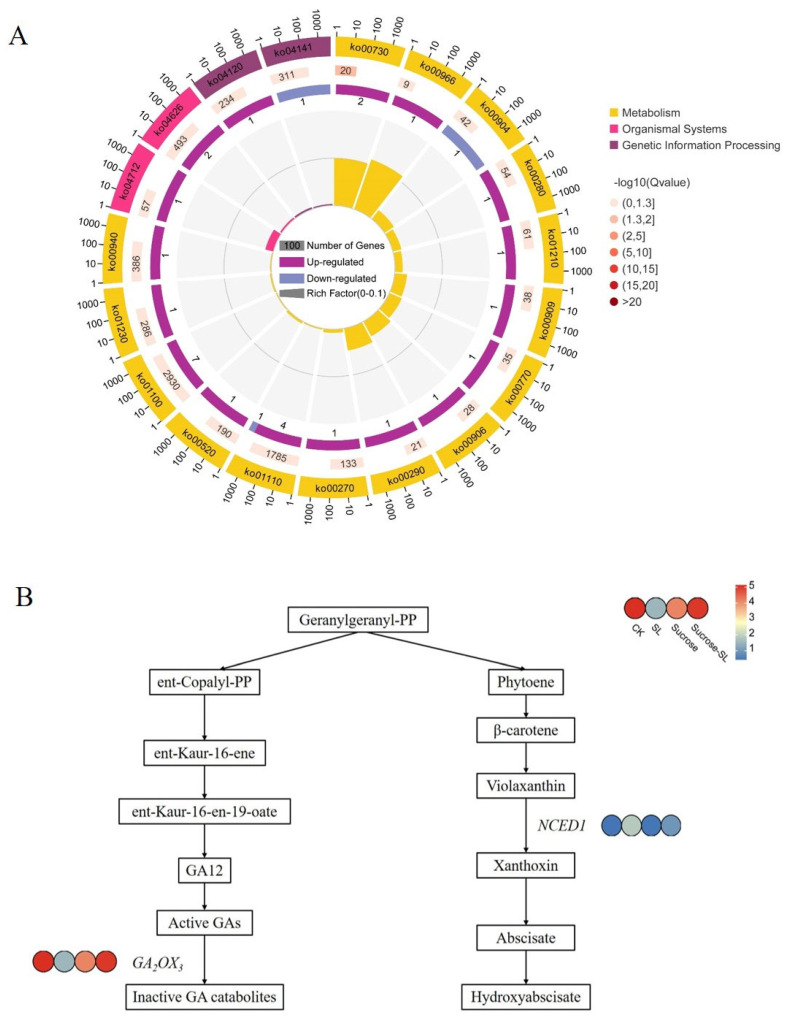
The key KEGG pathway in CK vs. SL group. (**A**) KEGG pathway analysis of CK vs. SL group; (**B**) Carotenoid biosynthesis and diterpenoid biosynthesis pathway in orchardgrass. The expression of genes encoding enzymes catalyzing corresponding biochemical reactions in different treatments are shown from blue to red, and the coloration scale and annotation are presented in the upright corner of this figure. *NCED1*, 9-cis-epoxycarotenoid dioxygenase; GA2OX3, gibberellin 2-beta-dioxygenase. The color from blue to red represents the expression from low to high.

The GO analysis of DEGs was enriched in the thiamine metabolic process (GO:0042723), oxazole or thiazole biosynthetic process (GO:0018131), establishment of localization (GO:0051234), water transmembrane transporter activity (GO:0005372), and amide transmembrane transporter activity (GO:0042887) in the CK vs. SL group (Figure S1). The KEGG pathway of this group was mainly enriched in metabolism pathways, such as thiamine metabolism (ko00730), glucosinolate biosynthesis (ko00966), carotenoid biosynthesis(ko00906), and diterpenoid biosynthesis (ko00904) (Figure 3A). Moreover, the plant–pathogen interaction of the organismal system (ko04626) is also enriched in this group. The pathway of thiamine metabolism (ko00730), diterpenoid biosynthesis (ko00904), and plant–pathogen interaction (ko04626) was upregulated according to the results of GSEA in CK vs. SL group (Figure S2). In these pathways, carotenoid biosynthesis and diterpenoid biosynthesis were very important for the process of tiller development. Among which, *NCED1* (*9-cis-epoxy-carotenoid dioxygenase1*) in the carotenoid biosynthesis pathway was upregulated, and *GA2OX3* (*gibberellin 2 beta-dioxygenase*, *DG6C01116*) in the diterpenoid biosynthesis pathway was downregulated (Figure 3B). *MYB73* (*DG3C02970*/*DG2C02624*) in plant–pathogen interaction was upregulated, which promotes the suppression of plant-defense responses (Figure S3). The expression of *CCD8* (*p*-value < 0.05) in SL biosynthesis decreased 1.4-fold, which contributed to the production of fewer SLs. In summary, *NCED1*, *GA2OX3*, *CCD8*, and *MYB73* might play important roles in tiller bud growth when SL is applied.

### 2.4. GO and KEGG Pathway Analysis of CK vs. Sucrose Group and CK vs. SL+Sucrose Group

**Figure 4:**
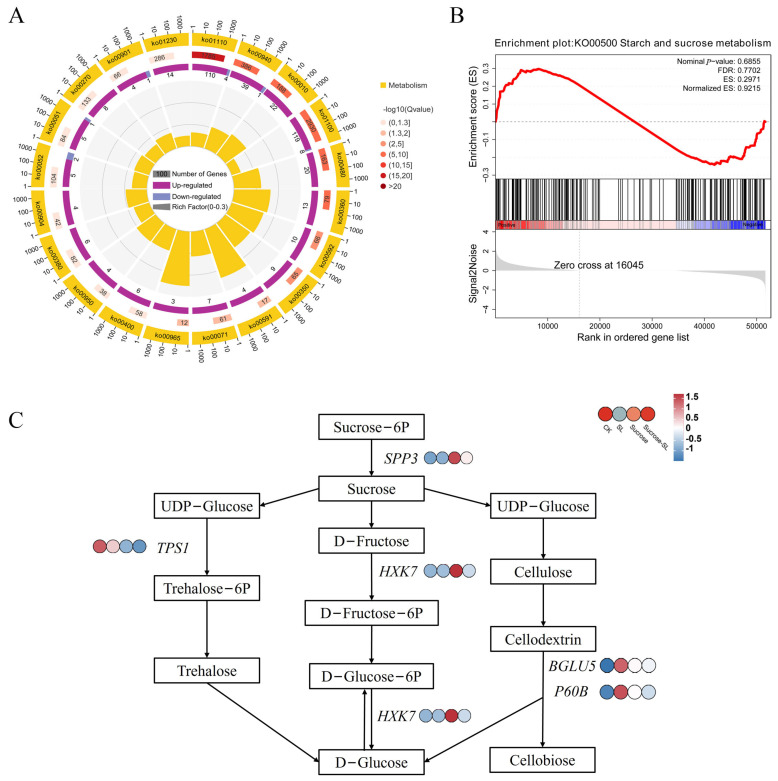
The key pathway in CK vs. Sucrose group. (**A**) KEGG enrichment analysis; (**B**) GSEA analysis; (**C**) The part of starch and sucrose metabolism pathway in CK vs. Sucrose group. TPS1 represents Trehalose-6-phosphate synthase; SPP3 represents sucrose-6-phosphatase; HXK7 represents hexokinase; BGLU5 and P60B represent beta-glucosidase. The color from blue to red represents the expression from low to high.

The GO analysis of DEGs was enriched in the metabolic process and catalytic activity, such as the phenylpropanoid metabolic process (GO:0009698), phenylpropanoid biosynthetic process (GO:0009699), regulation of proteolysis (GO:0030162), and regulation of peptidase activity (GO:0052547) in the CK vs. Sucrose group (Figure S4). The KEGG pathway of this group was significantly enriched in metabolism pathways, such as metabolic pathways (ko01100), glutathione metabolism (ko00480), and diterpenoid biosynthesis (ko00904) (Figure 4A). The pathway of starch and sucrose metabolism (ko00500) in metabolic pathways was upregulated according to the results of GSEA in the CK vs. sucrose group (Figure 4B). This indicates that sucrose is very important for the process of tiller development. Among them, *TPS1* (*trehalose-6-phosphate synthase*) in starch and sucrose metabolism was downregulated, and *SPP3* (*sucrose-phosphatase 3*), *HXK7* (*hexokinase-7*), *BGLU5* (*beta-glucosidase 5*) and *P60B* (*beta-D-glucosidase*) in it was upregulated in the CK vs. sucrose group (Figure 4C). Moreover, *GA2OX3* (*DG5C04245*) in diterpenoid biosynthesis increased 1.5-fold in this group. Interestingly, *GA2OX3* (*DG6C01116*) was downregulated in the CK vs. SL group, and *GA2OX3* (*DG5C04245*) was upregulated in the CK vs. Sucrose group, which verified that different *GA2OX3* types can be promoted by sucrose and repressed by SL. This plays an important role in the interaction of SL and sucrose to regulate tillering. *D3* in the SL signal transduction pathway increased 1.0-fold in this group, while *D3* is vital to the response of SL and sucrose. In total, *TPS1*, *HXK7*, *GA2OX3*, and *D3* might regulate tiller development by correlative sucrose pathways.

### 2.5. GO and KEGG Pathway Analysis of CK vs. SL+Sucrose Group

The GO analysis of DEGs was enriched in phenylpropanoid metabolic process (GO:0009698), phenylpropanoid biosynthetic process (GO:0009699), L-phenylalanine metabolic process (GO:0006558), arabinose metabolic process (GO:0019566), aromatic amino acid family metabolic process (GO:0009072) in CK vs. SL+Sucrose group (Figure S5). The KEGG pathway of this group was significantly enriched in phenylpropanoid biosynthesis (ko00940), biosynthesis of secondary metabolites (ko01110), phenylalanine metabolism (ko00360), metabolic pathways (ko01100), glutathione metabolism (ko00480) (Figure S6).

### 2.6. The Core Genes Involved in the Interaction of SL and Sucrose

**Figure 5:**
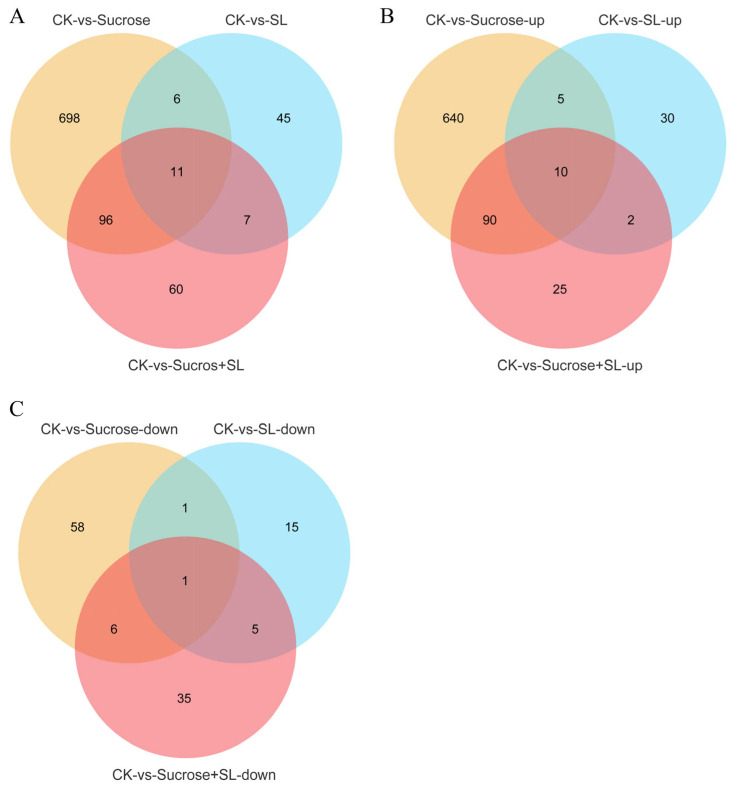
Venn diagrams of differentially expressed genes based on transcriptome sequencing. (**A**) all DEGs in three groups; (**B**) Upregulated DEGs in three groups; (**C**) Downregulated DEGs in three groups.

A total of 51 genes were differentially expressed in the CK vs. SL group, but none of them were DEGs in the CK vs. Sucrose+SL group (Figure 5A). Among them, the expressions of *DREB1H*, *DREB1B*, *NCED1*, *THI1-2*, and *MYB73* were significantly upregulated, and *SWEEET15* and *GA2OX3* were remarkably downregulated in the CK vs. SL group. Interestingly, there was virtually no difference in these gene expressions in the CK vs. SL+Sucrose group, which revealed that the variation of these gene expressions was repressed by the supplied sucrose. Exogenous sucrose can suppress the change in these gene expressions caused by SL and, therefore, release the inhibition of tiller bud development due to SL. A total of 704 genes were DEGs in the CK vs. Sucrose group but not DEGs in the CK vs. SL+Sucrose group (Figure 5A). Among them, the expression of *HOX18* (*DG2C02050*) and *ERF110* (*DG6C01367*) was significantly upregulated, and *NRT2.1* (*DG3C00190*) and *COL5* (*constans-like*) were remarkably downregulated in the CK vs. Sucrose group. However, the expression of these genes was virtually no different to the CK vs. SL+Sucrose group, which revealed that the change in these gene expressions was repressed by the supplied SL. In addition, there were 10 upregulated genes (*DRMH1*) and one downregulated gene (*PIP2-3*) in three groups (CK vs. SL, CK vs. Sucrose, and CK vs. SL+Sucrose group) (Figure 5B,C). There were five upregulated genes and one downregulated gene (*COL5*) in the CK vs. SL and CK vs. Sucrose group (when either exogenous sucrose or SL, but not both, was applied) (Figure 5B,C). *DRMH1* was both upregulated when exogenous sucrose or SL or both was applied, but *COL5* was only downregulated when either exogenous sucrose or SL, but not both, was applied, which indicates that *DRMH1* and *COL5* might serve in other unknown regulatory pathways.

### 2.7. PPI of the Core Genes Involved in the Interaction of SL and Sucrose

**Figure 6:**
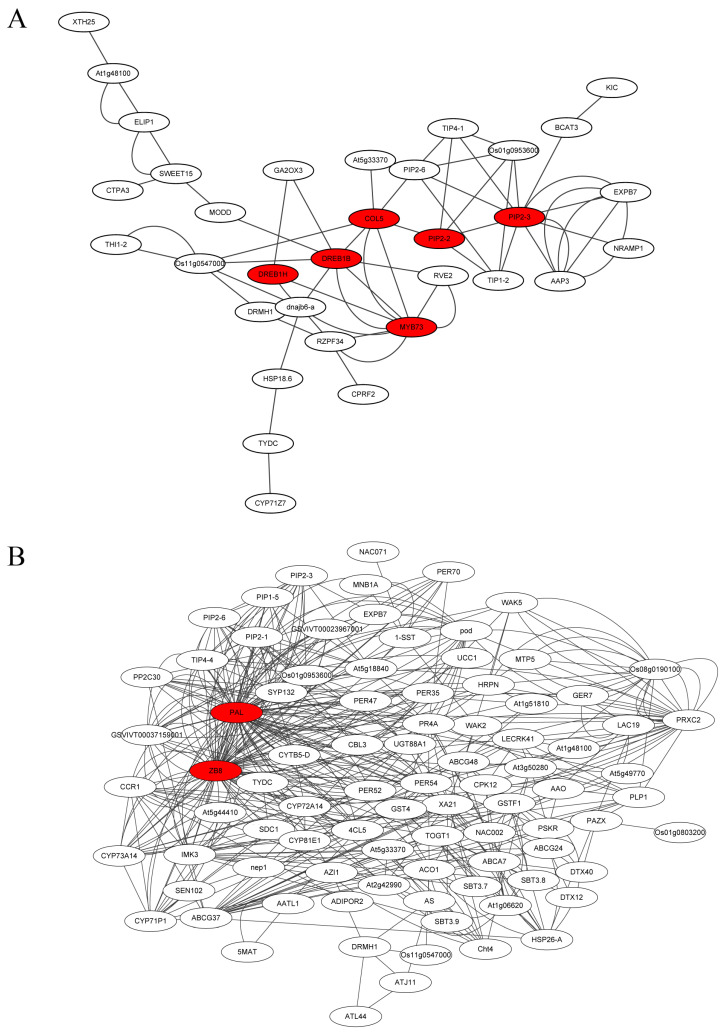
Protein–protein interaction (PPI) network. (**A**) The PPI of CK vs. SL group; (**B**) The PPI of CK vs. SL+Sucrose group. A solid black line represents an interaction.

The function of core genes involved in the interaction of SL and sucrose was further analyzed by the protein–protein interaction. The PPI of the CK vs. SL group revealed that COL5 could interact with MYB73, DREB1B (hydration-responsive element binding), and PIP2-2 (plasma membrane intrinsic protein), and PIP2-2 might interact with PIP2-3 (Figure 6A). In the CK vs. Sucrose group, the PPI predicts that COL5 may combine with HOX12, and D3 could interact with JUB1 (jungbrunnen 1), COL5, WRKY6, and NAC048 (Figure S7). Based on the results of PPI, it indicated that PAL (phenylalanine ammonia-lyase) and ZB8 (phenylalanine ammonia-lyase-like) would be the hub gene in CK vs. SL+Sucrose group (Figure 6B). It was reported that D3 plays a significant role in the interaction of SL and sucrose. Therefore, COL5 could be a key gene of the network of the interaction of SL and sucrose, which linked D3 with HOX12, MYB73, DREB1B, and PIP2-2. This study found some possible reciprocal genes of these candidate genes in each comparison by PPI, and it might provide some clues for subsequent mechanistic studies. 

### 2.8. The Predictable Interaction Mechanism of miRNA and mRNA by Association Analysis

**Figure 7:**
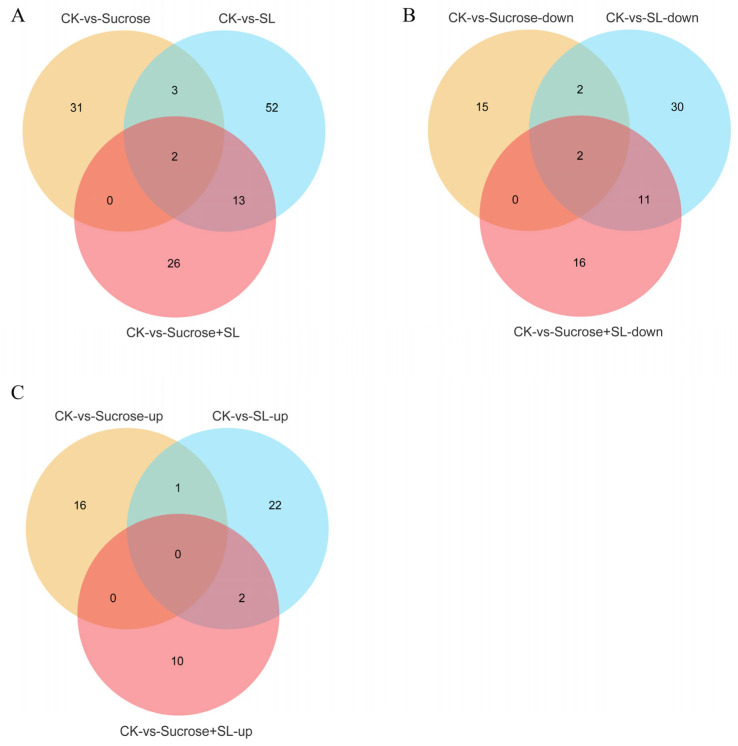
Venn diagrams of differentially expressed miRNA. (**A**) All DE miRNAs in three groups; (**B**) Downregulated DE miRNAs in three groups; (**C**) Upregulated DE miRNAs in three groups.

**Figure 8:**
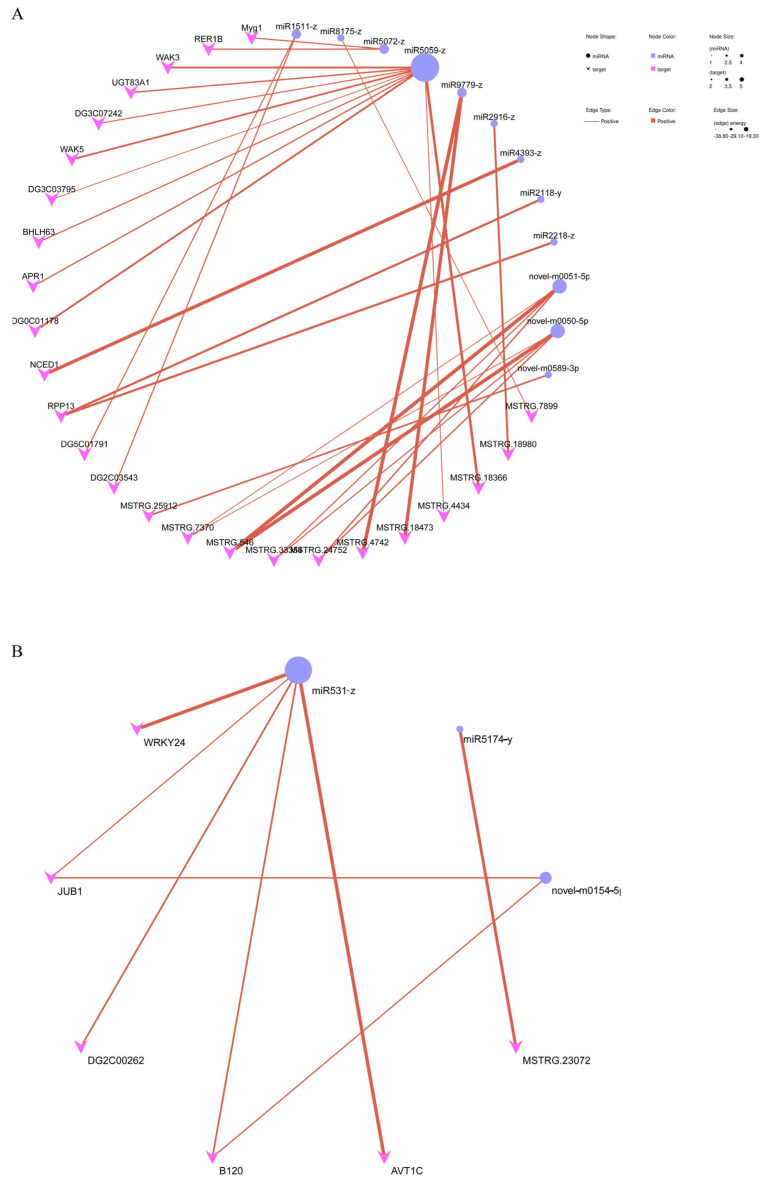
The network of DE miRNAs and their target genes in (**A**) CK vs. SL group and (**B**) CK vs. Sucrose group. A solid red line represents an interaction.

The function of miRNA in regulating tiller formation remains largely unknown. We also performed a miRNA analysis to reveal the mechanism of tiller formation in orchardgrass by applying the exogenous sucrose, SL, or both. The maximum number of differentially expressed miRNAs (DE miRNAs) was in the CK vs. SL group (Figure 7A and Table S6). Two DE miRNAs (miR172-x and novel-m0109-5p) were downregulated in three comparisons, which could be key miRNAs to respond to SL, sucrose application, or both (Figure 7B, Tables S6–S8). Novel-m0285-5p and novel-m0496-3p were upregulated in the CK vs. SL and CK vs. SL+Sucrose groups, which could be key miRNAs to respond to SL instead of sucrose (Figure 7C). Moreover,13 DE miRNA was differentially expressed in the CK vs. SL and CK vs. SL+Sucrose groups, which might be regulated by SL, such as miR171-x, miR7782-y, and so on (Figure 7A). The KEGG of DE miRNAs in the CK vs. SL group mainly enriched the spliceosome, and the KEGG of DE miRNAs in the CK vs. Sucrose group mainly enriched in phosphonate and phosphinate metabolism, and that of the CK vs. SL+Sucrose group enriched phenylpropanoid biosynthesis (Figure S8). The target genes of DE miRNAs in the CK vs. SL group were mainly enriched in carotenoid biosynthesis and nitrogen metabolism, and those of the CK vs. Sucrose group mainly enriched the MAPK signaling pathway (Figure S9). In the CK vs. SL group, miR5059-z has ten predictable target genes, and *NCED1* could be the target of miR4393-z according to mRNA and miRNA correlation analysis. In the CK vs. Sucrose group, miR531-z has five predictable target genes, including *JUB1* (*DG4C05239*) and *WRKY24* (*DG5C04598*) (Figure 8).

## 3. Discussion

Orchardgrass is one of the best perennial forage grasses in the world [30]. It is widely used as an ideal grass forage for artificial mixed grass or forage production and can also be used for stone desertification management in southwest China [31]. As a perennial forage grass, the regeneration capacity, nutrient yield, and seed yield of orchardgrass are partially or fully dependent on tillering. Moreover, orchardgrass is a good species to study the tillering mechanism of perennial forage because of the prominent tillering ability of orchardgrass among crops. However, the mechanism of tillering in orchardgrass is still largely unknown, and the role of sucrose as a new tiller regulator and SLs in the regulation of orchardgrass tillering is still to be studied. Therefore, we performed a transcriptome and miRNA analysis to reveal the potential RNA mechanism of tiller formation under strigolactone and sucrose treatment in orchardgrass. In our experiment, the development of tiller buds was repressed by the exogenous application of SL analog GR24 (Figure 1). It is indisputable that SL does inhibit the growth of tiller buds in orchardgrass. However, we found the GAs might be increased for downregulated *GA2OX3* (*DG6C01116*) with GR24 applied, which participated in delaying tiller bud development. Previous studies indicated the existence of physiological cross-talk between SLs and GAs in not only the regulation of SL biosynthesis but also tiller bud outgrowth and plant elongation [32]. It is suggested that *GA2OX3* (*DG6C01116*) could be a key gene in the regulation of tiller bud development by SLs and GAs. Moreover, our study found that exogenous application of GR24 may cause a rise in ABA biosynthesis because of the upward expression of *NCED1*. The increased ABA was involved in hindering tiller bud development according to the phenotype change in our study (Figure 1), which is consistent with previous reports [10,33]. It was reported that *CCD8*/*D10* had a great impact on the feedback of SL biosynthesis [6,34,35]. Additionally, we found that the possible elevated ABA in turn inhibits the expression of *CCD8*/*D10* (*p*-value < 0.05), which resulted in the decreasing biosynthesis of SLs in vivo. *CCD8*/*D10* could be a vital gene in the regulation of tiller bud development in orchardgrass by SL and ABA. This indicated that SL inhibits the development of tiller buds, and its synthesis gene *CCD8* plays an important role in tiller bud formation in orchardgrass. 

TPS is a key enzyme in starch and sucrose metabolism [36,37]. In our research, *TPS1* was downregulated, and *SPP3*, *HXK7*, *BGLU5*, and *P60B* were upregulated in starch and sucrose metabolism in the CK vs. sucrose group. In total, the pathway of starch and sucrose metabolism (ko00500) was upregulated according to the results of GSEA in the CK vs. sucrose group (Figure 4B). These suggested that *SPP3*, *HXK7*, *BGLU5*, and *P60B* might be crucial regulators to respond to sucrose in orchardgrass. In vascular plants, disaccharide sucrose is one of the high-level sugars that provides a major source of carbon and energy [38]. It is supposed that rapid tiller bud development is partly due to excess energy supply caused by the exogenous application of sucrose. The level of GAs may be decreased because of increasing *GA2OX3* (*DG5C04245*) caused by exogenous sugar application, which releases the inhibition of tiller bud growth in orchardgrass. This indicates that GA/GA2OX3 might be a key factor in regulating tiller bud development and responding to exogenous sugar application. Interestingly, *GA2OX3* (*DG6C01116*) was downregulated in the CK vs. SL group, and *GA2OX3* (*DG5C04245*) was upregulated in the CK vs. Sucrose group, which verified that different *GA2OX3* types can be promoted by sucrose or repressed by SL, and GA2OX3 could play an important role on the response to SL and sucrose to regulate tillering in orchardgrass. D3 plays a crucial role in the interaction of SL and sucrose in rice [17]. Additionally, we found that exogenous sugar application induced the rising D3 expression in the SL signal transduction pathway (Figure S10), which supported that D3 is vital to the response of SL and sucrose in orchardgrass. However, the rising D3 expression during exogenous sugar application is contradictory to the results in rice, and the reason for this may be different response patterns in different species. Alternatively, different sucrose treatment times may result in different response patterns. Therefore, sucrose might regulate tillering in orchardgrass by the GA pathway.

*DREB1H*, *DREB1B*, *NCED1*, *THI1-2*, and *MYB73* were significantly upregulated, and *SWEEET15* and *GA2OX3* (*DG6C01116*) were remarkably downregulated in the CK vs. SL group. *DREB1H*, *DREB1B*, *NCED1*, *THI1-2*, and *MYB73* were induced by GR24, which could inhibit tiller bud growth. *SWEEET15* and *GA2OX3* (*DG6C01116*) were repressed by GR24, which could promote the development of tiller buds. However, there were no differential-expression genes in the CK vs. SL+Sucrose group. These revealed that these genes were significant in the interaction of SL and sucrose. We presumed that exogenous sucrose could suppress the change in their expression caused by SL and thus release the inhibition of the tiller bud development due to SL. The significantly upregulated *HOX18* (*DG2C02050*) and *ERF110* (*DG6C01367*) and remarkably downregulated *NRT2.1* (*DG3C00190*) and *COL5* are DEGs in the CK vs. Sucrose group but not in the CK vs. SL+Sucrose group. *HOX18* (*DG2C02050*) and *ERF110* (*DG6C01367*) were induced by sucrose, which could enhance tiller bud growth. *NRT2.1* (*DG3C00190*) and *COL5* were repressed by sucrose, which could inhibit the development of tiller buds. The change in these gene expressions caused by sucrose was repressed by the supplied SL. This indicates that exogenous sucrose can inhibit tiller bud development due to SL, but exogenous SL still can inhibit the growth of tiller buds caused by sucrose. In addition, *DRMH1* was upregulated when exogenous sucrose or SL or both was applied, but *COL5* was only downregulated when either exogenous sucrose or SL, but not both, was applied, which indicated that *DRMH1* and *COL5* might serve in other unknown regulatory pathways. Overall, *DREB1H*, *DREB1B*, *NCED1*, *THI1-2*, *MYB73*, *NRT2.1* (*DG3C00190*), and *COL5* could be involved in inhibiting the development of tiller buds in orchardgrass. *SWEEET15*, *GA2OX3* (*DG6C01116*), *HOX18* (*DG2C02050*), and *ERF110* (*DG6C01367*) could promote the development of tiller bud in orchardgrass.

**Figure 9:**
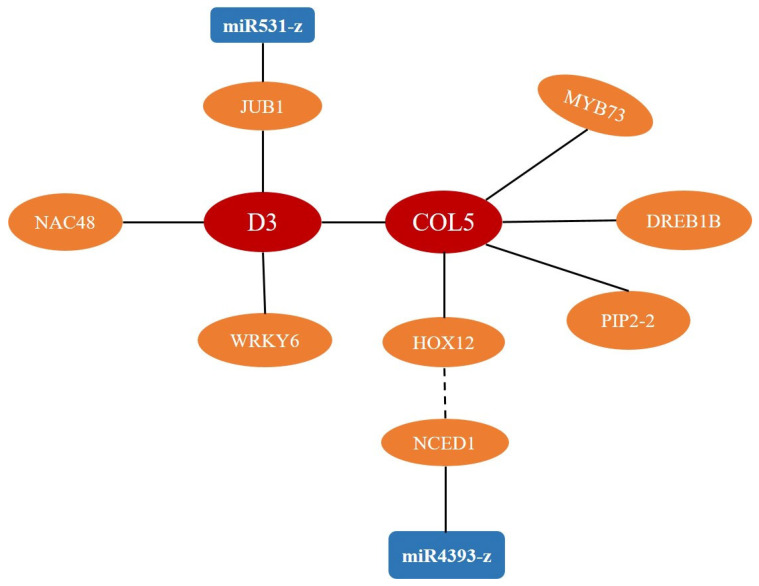
A predictable model of tillering in orchardgrass based on the PPI results and association analysis of miRNA and mRNA. A solid black line represents a potential interaction based on results.

Our study also found some possible reciprocal genes of these candidate genes in each comparison by PPI, and this might provide some clues for subsequent mechanistic studies. PAL and ZB8, the hub proteins in the CK vs. SL+Sucrose group (Figure 7B), both function as an enzyme of phenylalanine ammonia-lyase-like, which is consistent with the KEGG results (enriched in phenylalanine metabolism (ko00360)). This indicates that tiller bud development is very close to the phenylalanine metabolism when SL and sucrose are present. Recently, it was reported that D3 plays a significant role in the interaction of SL and sucrose [17]. In PPI, we found that COL5 linked D3 with HOX12 in the CK vs. Sucrose group (Figure S7), i.e., SL promotes the biosynthesis of ABA by *HOX12* and *NCEDs* [33]. This indicates that COL5 could be involved in SL signal transduction and ABA biosynthesis. TPS1, a key catalyzing enzyme in starch and sucrose metabolisms [39], might be an important regulator in sucrose response. In addition, TPS1, WRKY6, MYB73, DREB1B, and PIP2-2 were all involved in plant defense [40,41,42,43,44], which suggests that D3 and COL5 could also be an intermediate factor in responding to plant defense, indicating that the interaction of SL and sucrose still induces changes in plant defense. Therefore, D3 and COL5 could be two key genes in the network of the interaction of SL and sucrose (Figure 9). 

To date, a few miRNAs regulating tillering have been reported. We study the changes in small RNA under different exogenous treatments to explore the miRNA mechanism of tillering in orchardgrass. Among them, miR172-x and novel-m0109-5p are downregulated in three comparisons, which could be key miRNAs to respond to SL, sucrose application, or both. In particular, downregulated miR5059-z has ten predictable target genes in the CK vs. SL group (Figure 8A). This indicates that miR5059-z repressed by SL could be a vital factor in regulating the development of tiller buds, and its predictable target genes could play a negative role in regulating tillering development. *NCED1*, encoding a vital enzyme in ABA biosynthesis [45], could be the target of miR4393-z according to mRNA and miRNA correlation analysis (Figure 8A), and this indicates that downregulated miR4393-z might lead to the increase in *NCED1* to inhibit the tiller bud growth through the ABA pathway (Figure 9). Moreover, *JUB1 (DG4C05239*) is one target of miR531-z (downregulated) in the CK vs. Sucrose group (Figure 8B). Combined with the result of PPI, JUB1 can interact with D3. We assumed that downregulated miR531-z could lead to increasing JUB1, and subsequently, JUB1 interacted with D3 to regulate tillering by delaying the SL signal when sucrose was applied (Figure 9).

## 4. Materials and Methods

### 4.1. Plant Material and Treatments

A total of 76 tillers of the high-tillering orchardgrass (AKZ-NRGR667) selected from our previous study were grown in silica sand with quantitative half-strength Murashige & Skoog (½MS) solution supplied every day [29]. Sixty individual plants of similar sizes were randomly assigned to four independent groups of 15 plants. Among them, one group was selected as the control group, and the other three groups acted as treatment groups with rac-GR24 (an SL analog), sucrose, and rac-GR24+sucrose. Subsequently, different groups were grown on the solidified ½MS media (pH = 6.0) with different treatments for an hour. For the SL treatments, 1 μM rac-GR24 was used in all the experiments (Coolaber, Bejing, China) [17]. For the sucrose treatments, 4% sucrose was used in the relative treatment experiment. For the rac-GR24+sucrose treatments, 1uM rac-GR24 and 4% sucrose were used in this experiment. The plants of the control group were grown on ½MS media without 1 μM GR24 and sucrose. 

All materials were under the tillering stage and grown in a growth chamber, and it was a 22 °C/15 °C day/night temperature regime, a photoperiod of 14 h/10 h (day/night), and 70% relative humidity. Samples of shoot base from different treatments were simultaneously collected from biological replicates in a group after an hour of treatment, and samples from one biological replicate were mixed as a replicate. The collected samples were immediately frozen in liquid nitrogen and stored at −80 °C until RNA extraction and subsequent transcriptome sequencing.

Sixteen other similar-sized individual plants were randomly assigned to four independent groups of four replicates treated as described above at different times to observe the variation of phenotype. These plants were grown on ½MS media for 5 days with or without 1 μM GR24 or/and 4% sucrose.

### 4.2. RNA and miRNA-Seq Library Preparation and Sequencing

Total RNA was extracted using a Trizol reagent kit (Invitrogen, Carlsbad, CA, USA), and its quality and concentration were assessed by RNase-free agarose gel electrophoresis and Agilent 2100 Bioanalyzer. Subsequently, qualified RNA was enriched by Oligo (dT) beads and used to construct 12 cDNA libraries for RNA-seq. Then, the 12 cDNA library was sequenced using Illumina Novaseq6000 by Gene Denovo Biotechnology Co. (Guangzhou, China). For small RNA sequencing, the size of 18–30 nt RNAs was enriched by polyacrylamide gel electrophoresis (PAGE). The 3′ adapters were added, and the 36–48 nt RNAs were enriched. Then, the 5′ adapters were ligated to the RNAs, and these products were reverse transcribed by PCR amplification. Among them, the size of 140–160 bp PCR products were enriched and used to generate 12 cDNA libraries, which were sequenced using Illumina HiSeq Xten by Gene Denovo Biotechnology Co. (Guangzhou, China). The raw sequence data reported in this paper have been deposited in the Genome Sequence Archive (Genomics, Proteomics & Bioinformatics 2021) in National Genomics Data Center (Nucleic Acids Res 2022), China National Center for Bioinformation/Beijing Institute of Genomics, Chinese Academy of Sciences (GSA: CRA010605) that are publicly accessible at https://ngdc.cncb.ac.cn/gsa, accessed on 25 October 2023 [46,47]. 

### 4.3. Identification and Functional Annotation of Differentially Expressed Genes and miRNAs

The filtered clean reads were further used to assemble and calculate gene abundance (Table S1). Then, for RNA-seq, paired-end clean reads were mapped to the orchardgrass reference genome [48], and an FPKM value was calculated using RSEM 1.2.19 software. Differential-expression analysis of RNAs was analyzed by DESeq2 softwareV1.20.0 between two different groups [49]. The differentially expressed genes were set as the false discovery rate (FDR) < 0.05 and the absolute fold change ≥ 1. Gene-set enrichment analysis shows the significant differences of a set of genes in specific GO terms or KEGG pathways in two groups using the software GSEA V2.2.4 [50]. String v10 was used to generate protein–protein interaction network [51], where genes were determined as nodes and interactions as lines in a network. Additionally, the network was present as a picture of a core and hub gene biological interaction using Cytoscape (v3.7.1) software [52].

For small RNA sequencing, all the clean tags were aligned with the orchardgrass reference genome to clean fragments caused by mRNA degradation and aligned with small RNAs in the GeneBank database to remove rRNA, scRNA, snoRNA, snRNA, and tRNA. The remaining clean tags were aligned with small RNAs in the Rfam database (Release11.0) [53]. Generally, total miRNA was divided into existing miRNA, known miRNA, and novel miRNA. Known miRNAs were identified by searching and aligning with other species in the miRBase database [54]. The novel miRNA candidates were identified by the genome positions and hairpin structures of these unannotated tags, which were predicted by the software mirdeep2 [55]. The expression level of miRNA was calculated and normalized according to transcripts per million (TPM) based on their expression in each sample, and their differential-expression analysis between two different groups was performed by software edgeR 3.12.1. miRNAs with a fold change ≥ 1 and *p*-value < 0.05 in comparison were identified as significant DE miRNAs [56]. The target genes of DE miRNAs were predicted by the software patmatch, and then GO and pathway enrichment analysis of target genes was performed according to common standards [57].

### 4.4. Real-Time Quantitative RT-PCR

Samples were from 4.1, and the total RNA was extracted using HiPure plant RNA Mini Kit (MAGEN, Guangzhou, China) and reverse transcribed into first-strand cDNA using the MonScript™ RTIII All-in-One Mix with dsDNase (Monad, Wuhan, China). The miRNA was extracted using HiPure Universal miRNA Kit (MAGEN, Guangzhou, China) and reverse transcribed into first-strand cDNA using the MonScript™ miRNA First-Strand cDNA Synthesis Kit (Monad, Wuhan, China). qRT-PCR was conducted using MonAmp™ SYBR Green qPCR Mix (Monad); qRT-PCR reactions were 10 μL, including 5.0 μL of SYBR Green qPCR Mix, 0.5 μL of each primer, 3.0 μL of H_2_O, and 1 μL of cDNA samples at a 4-fold dilution and 1 μL of cDNA samples (miRNA) at a 10-fold dilution. *GAPDH* and *U6* were used as the internal reference gene [58]. The relative gene expression levels were evaluated using the 2^−∆∆Ct^ method [59,60]. Three replicates were conducted for each experiment. All primers were designed using Primer5 (Table S2).

## 5. Conclusions

Based on our research, we found that *D3*, *COL5*, *NCED1*, *HXK7*, miRNA4393-z, and miRNA531-z could be key factors to control tiller bud development in orchardgrass. Moreover, strigolactones might affect the ABA biosynthesis pathway to regulate the tiller bud development of orchardgrass, which may be related to the expression changes in miRNA4393-z, *NCED1*, and *D10*. miRNA531-z could be involved in the interaction of strigolactones and sucrose to regulate tillering. According to that, we provided a potential model of the molecular mechanism of tillering in orchardgrass, which will be further used to clarify the potential mechanism of tillering in orchardgrass and will be beneficial to improve the production and reproduction of crops.

## Data Availability

The raw sequence data reported in this paper have been deposited in the Genome Sequence Archive (Genomics, Proteomics & Bioinformatics 2021) at the National Genomics Data Center (Nucleic Acids Res 2022), China National Center for Bioinformation/Beijing Institute of Genomics, Chinese Academy of Sciences (GSA: CRA010605) that are publicly accessible at https://ngdc.cncb.ac.cn/gsa, accessed on 25 October 2023.

## Supplementary Materials

The following supporting information can be downloaded at https://www.mdpi.com/article/10.3390/ijms242115762/s1.
