# Supplementary figures and images for: Transcriptome Analysis Reveals the Potential Molecular Mechanisms of Tiller Bud Development in Orchardgrass
Source: Int J Mol Sci. 2023 Oct 30;24(21):15762. doi: 10.3390/ijms242115762 (PMC10650679; doi:10.3390/ijms242115762)

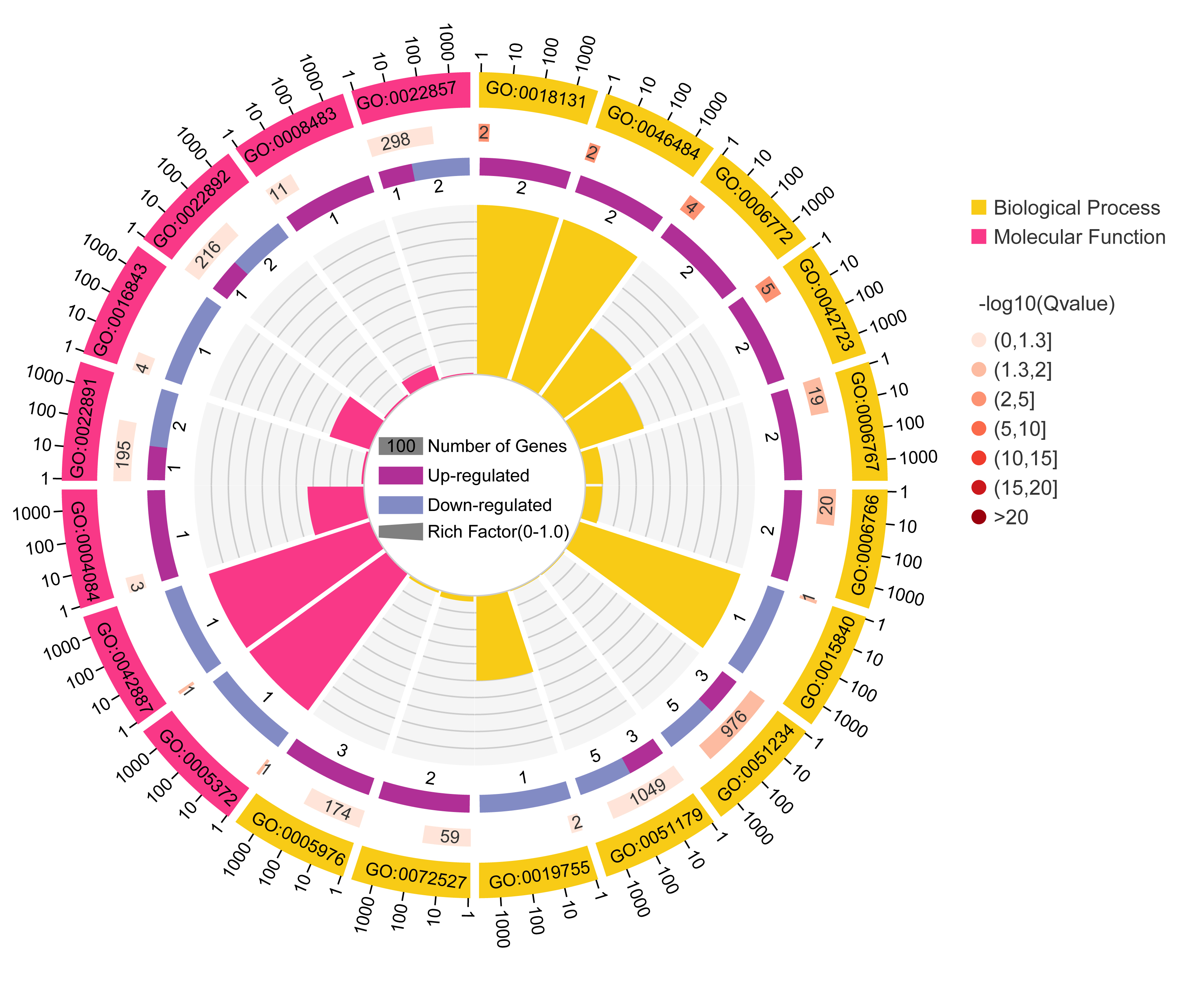

Supplement: Supplementary file 1 [file ijms-24-15762-s001.zip › Fig S1.jpg]

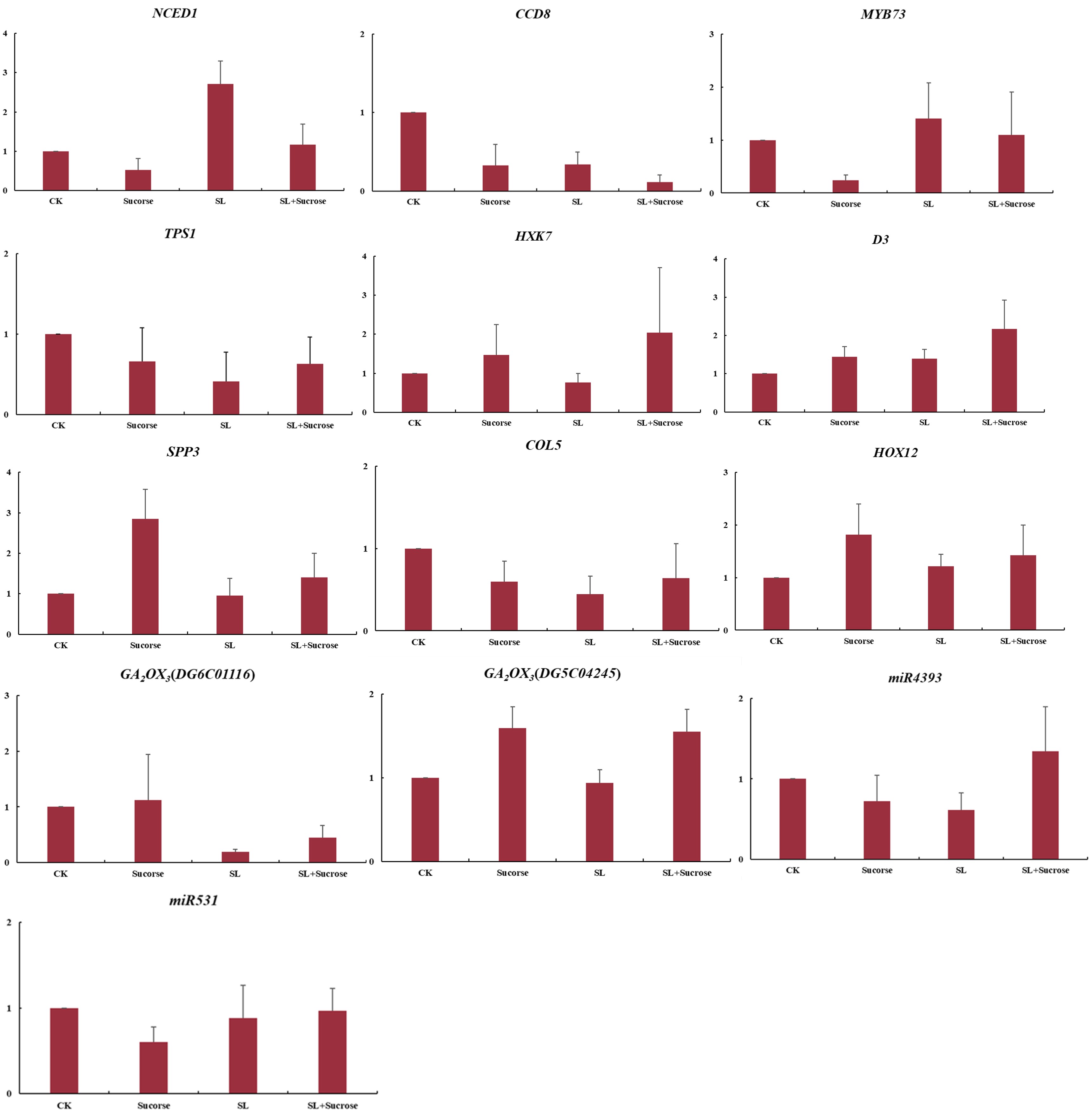

Supplement: Supplementary file 1 [file ijms-24-15762-s001.zip › Fig S10.jpg]

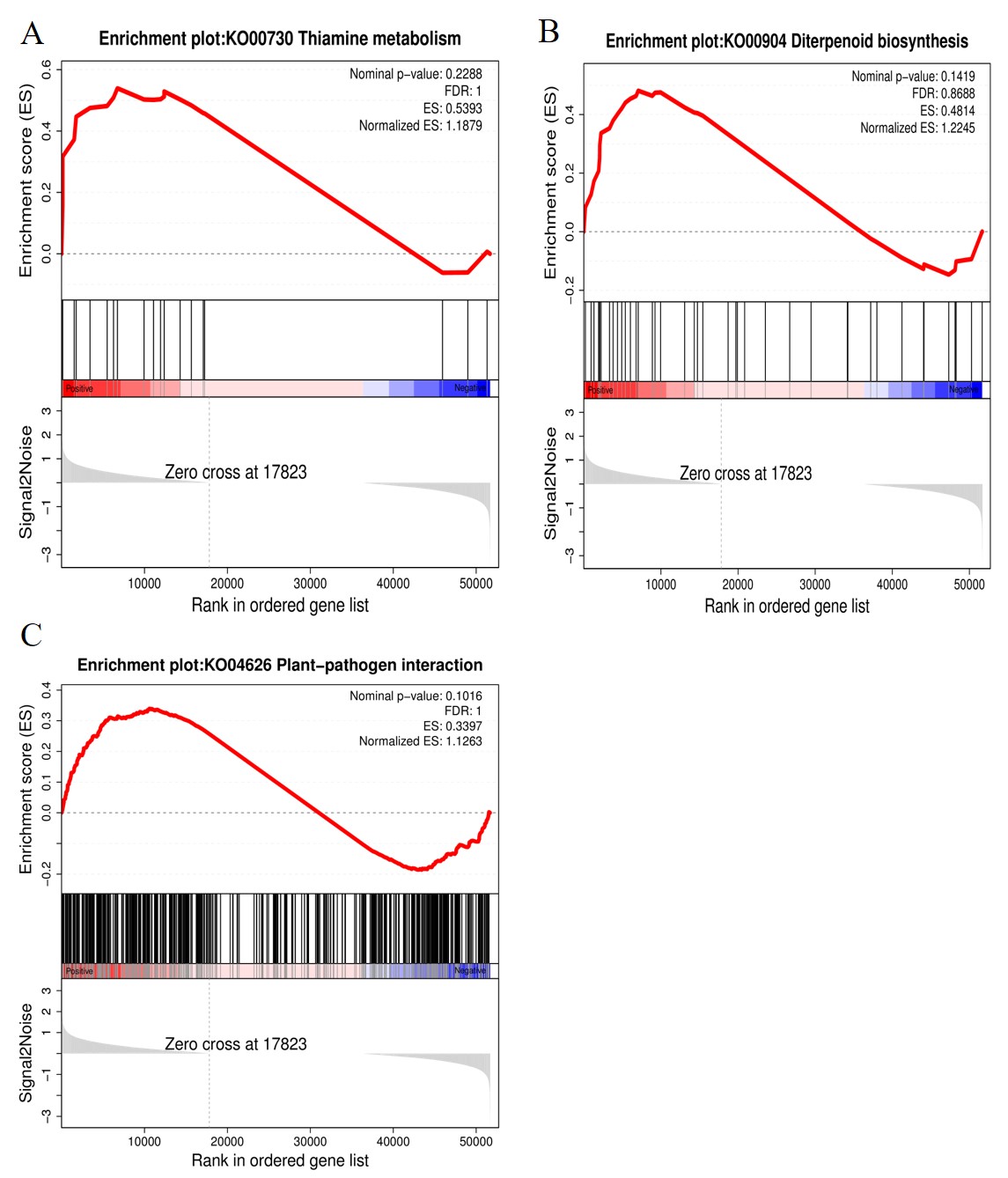

Supplement: Supplementary file 1 [file ijms-24-15762-s001.zip › Fig S2.jpg]

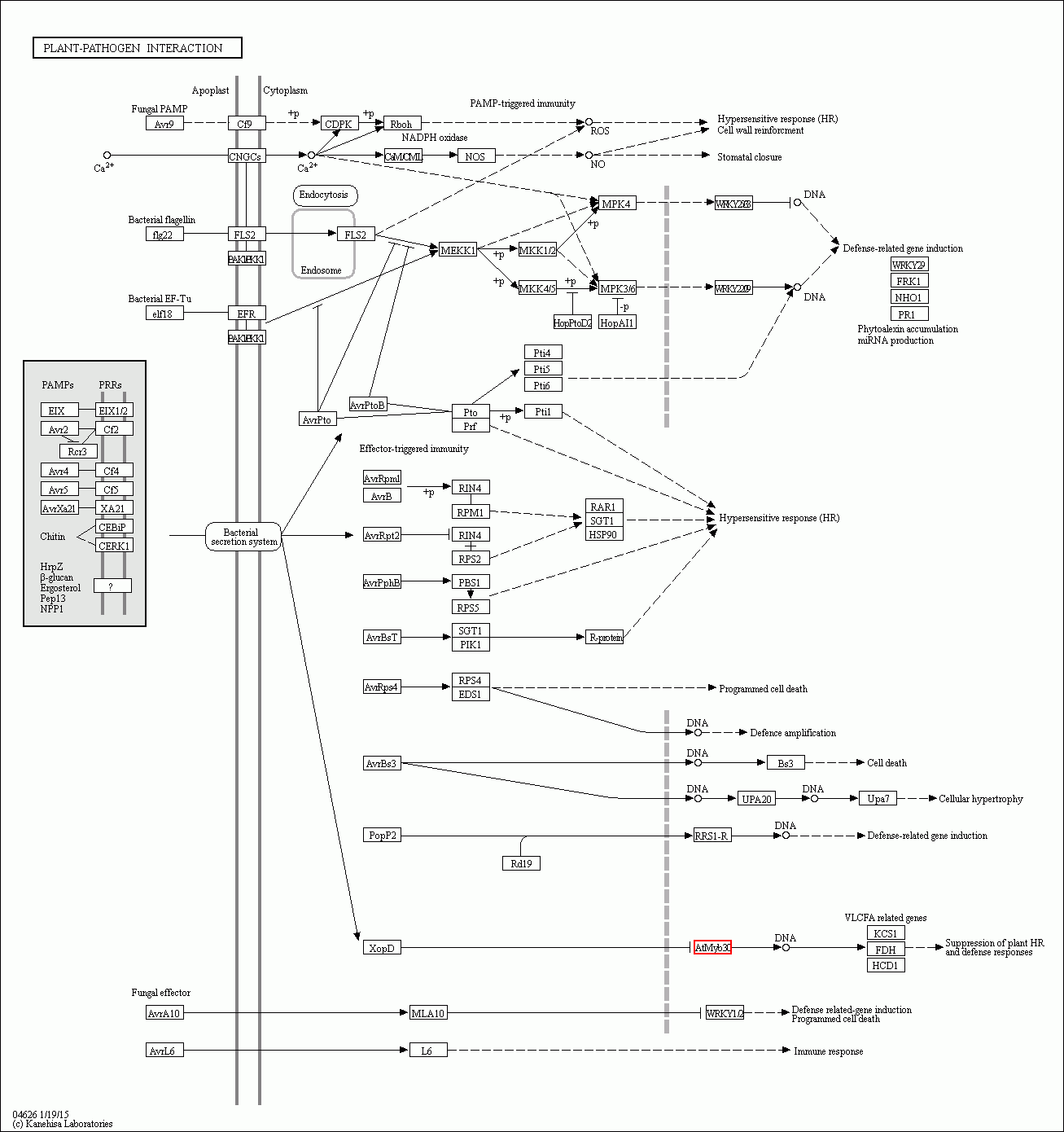

Supplement: Supplementary file 1 [file ijms-24-15762-s001.zip › Fig S3.png]

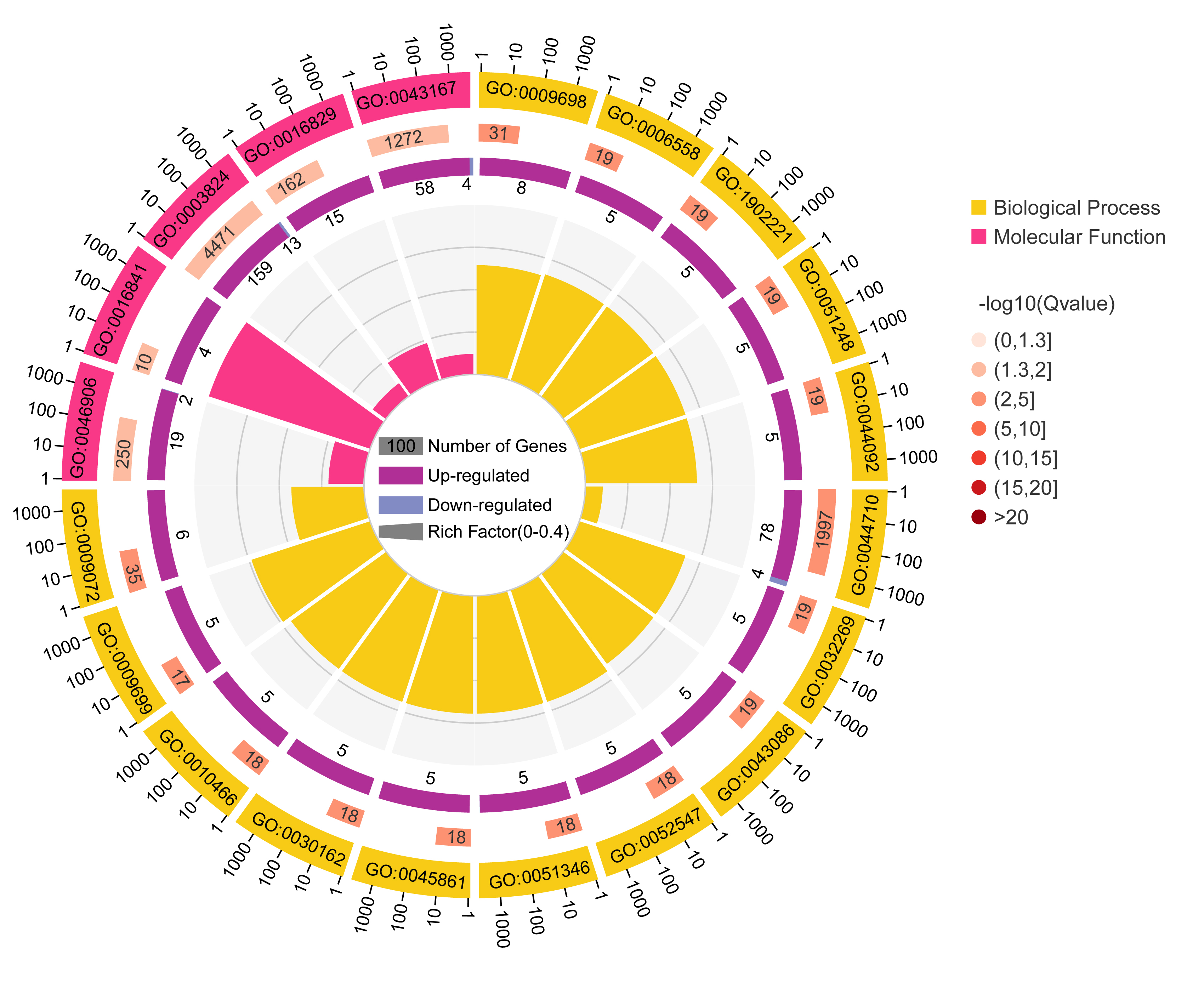

Supplement: Supplementary file 1 [file ijms-24-15762-s001.zip › Fig S4.jpg]

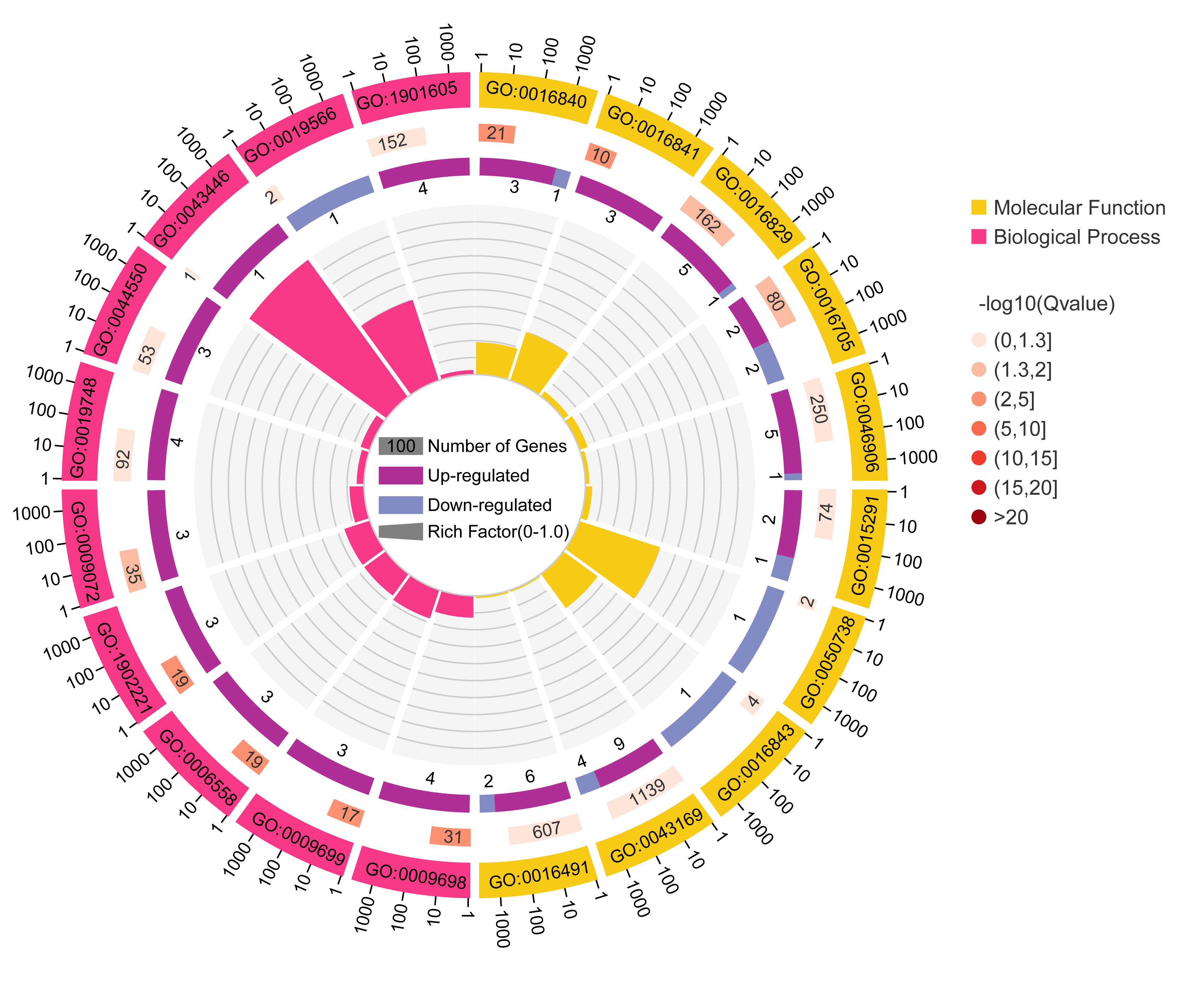

Supplement: Supplementary file 1 [file ijms-24-15762-s001.zip › Fig S5.jpg]

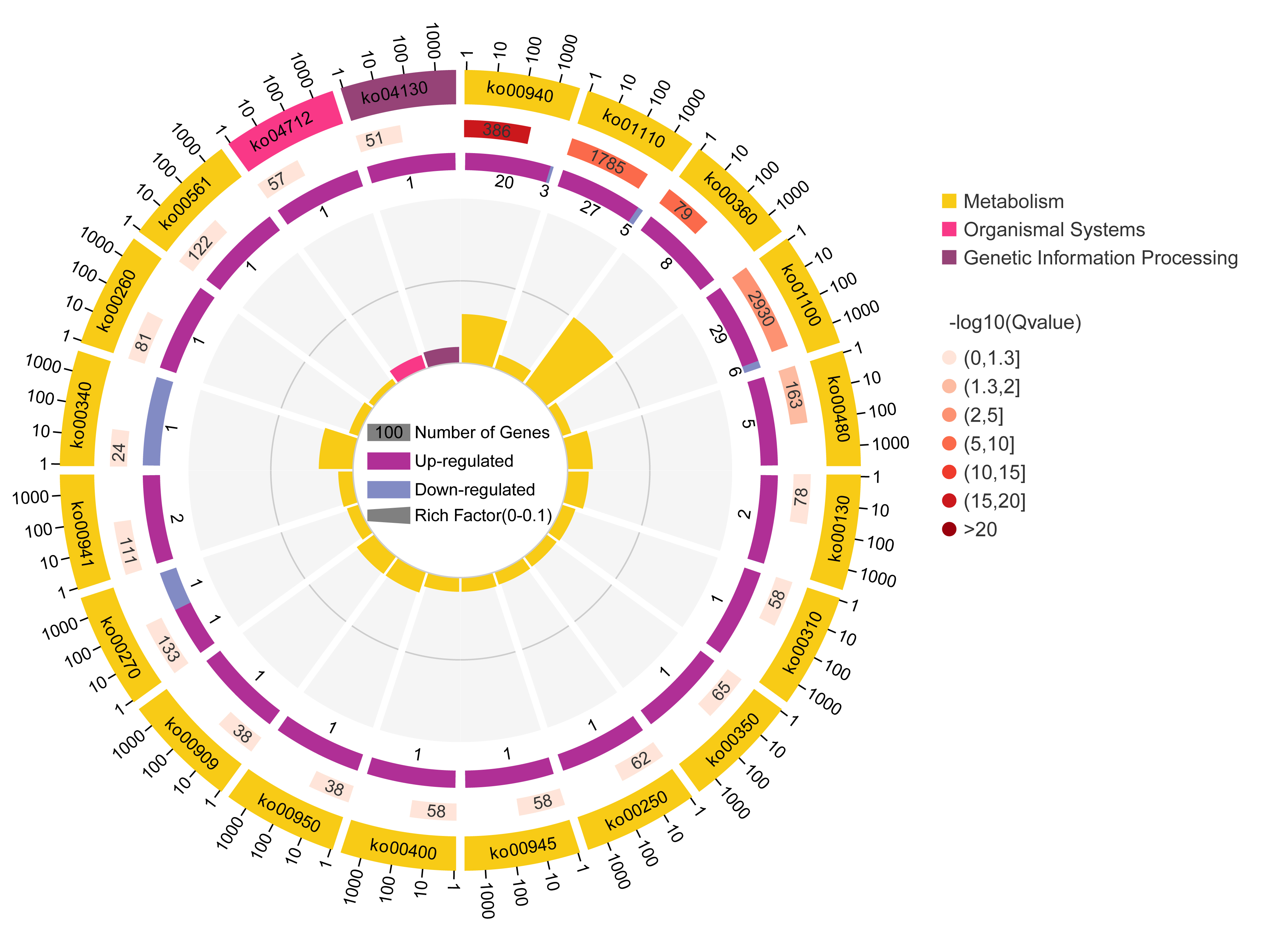

Supplement: Supplementary file 1 [file ijms-24-15762-s001.zip › Fig S6.jpg]

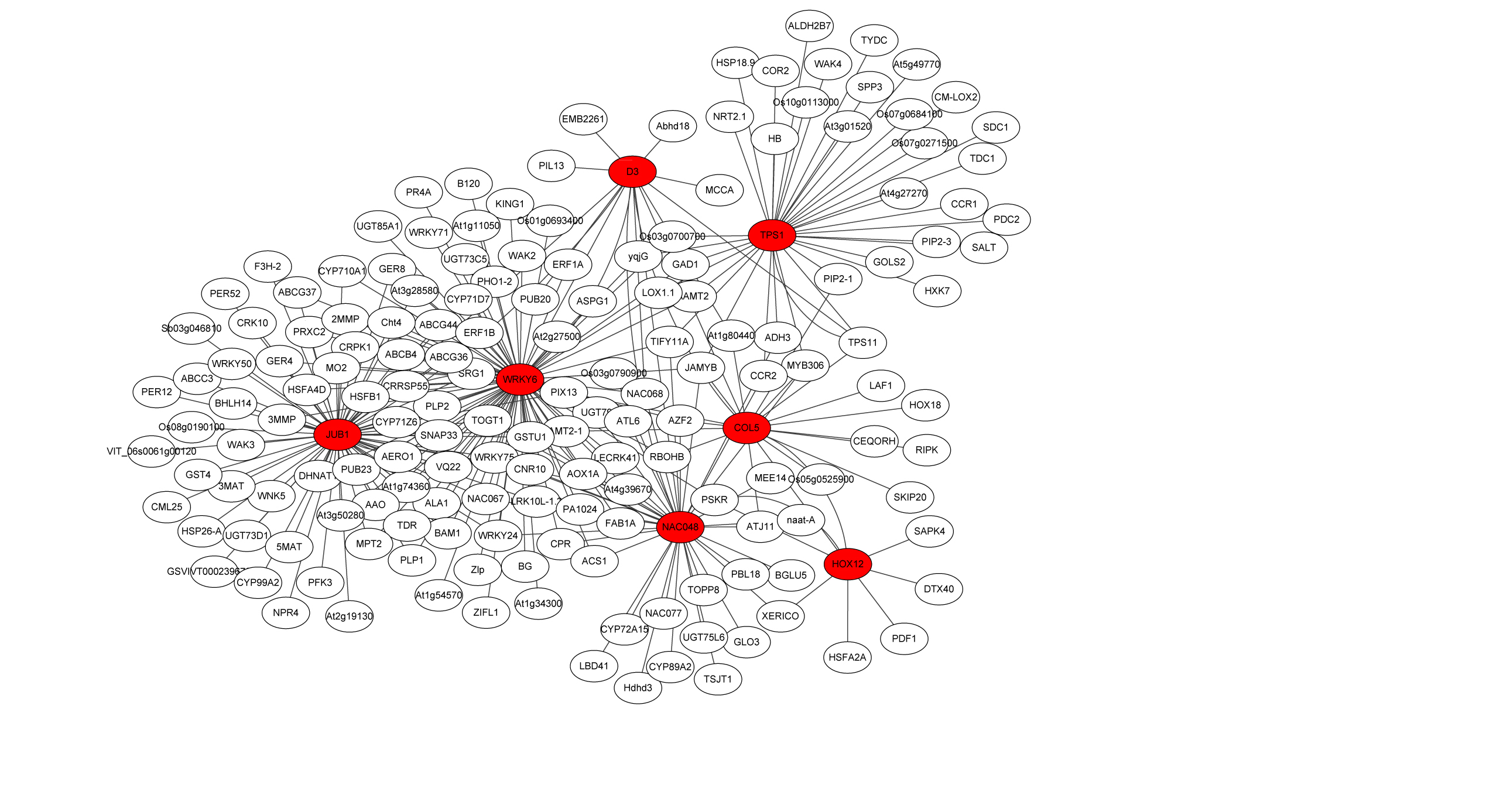

Supplement: Supplementary file 1 [file ijms-24-15762-s001.zip › Fig S7.jpg]

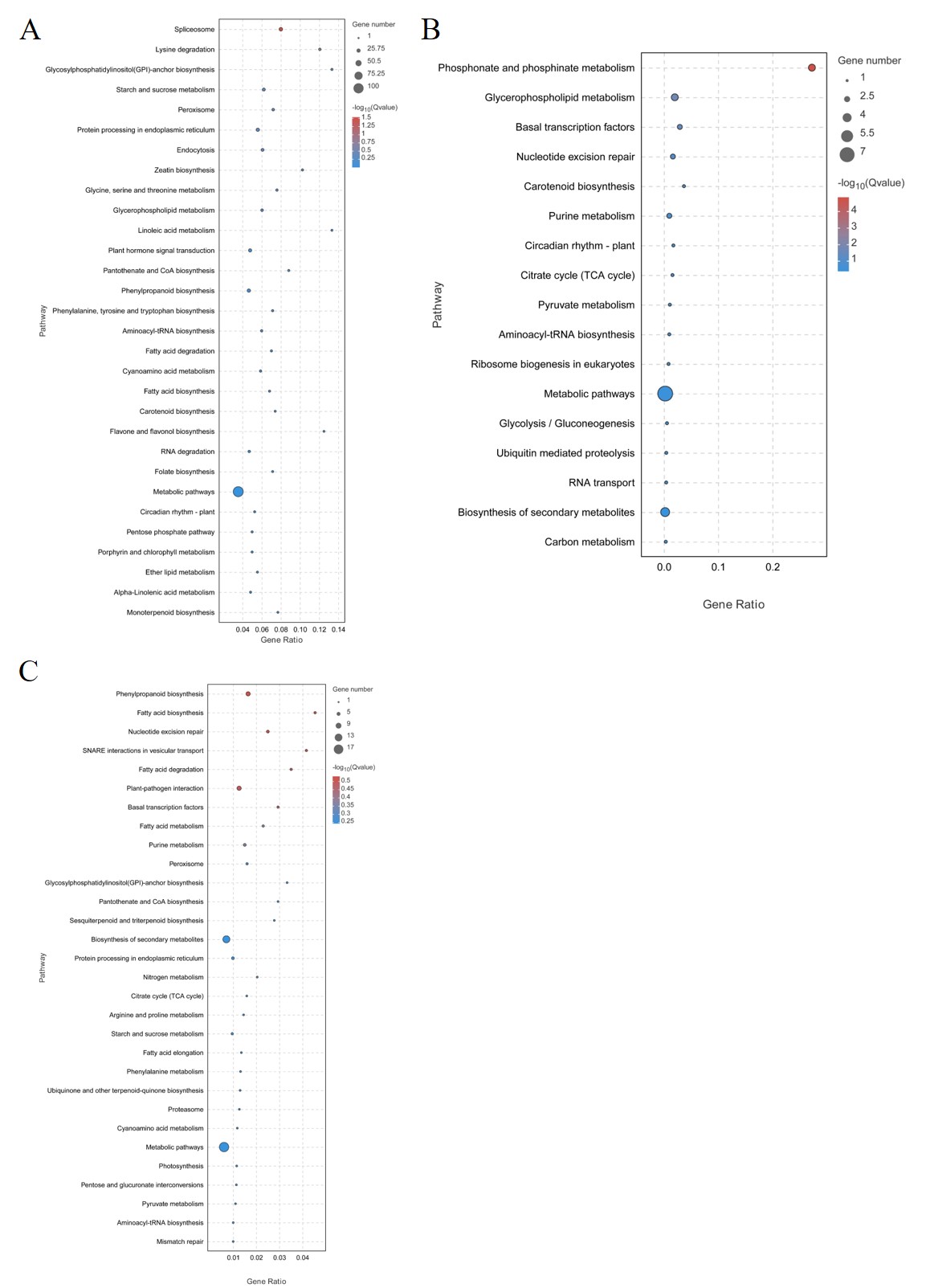

Supplement: Supplementary file 1 [file ijms-24-15762-s001.zip › Fig S8.jpg]

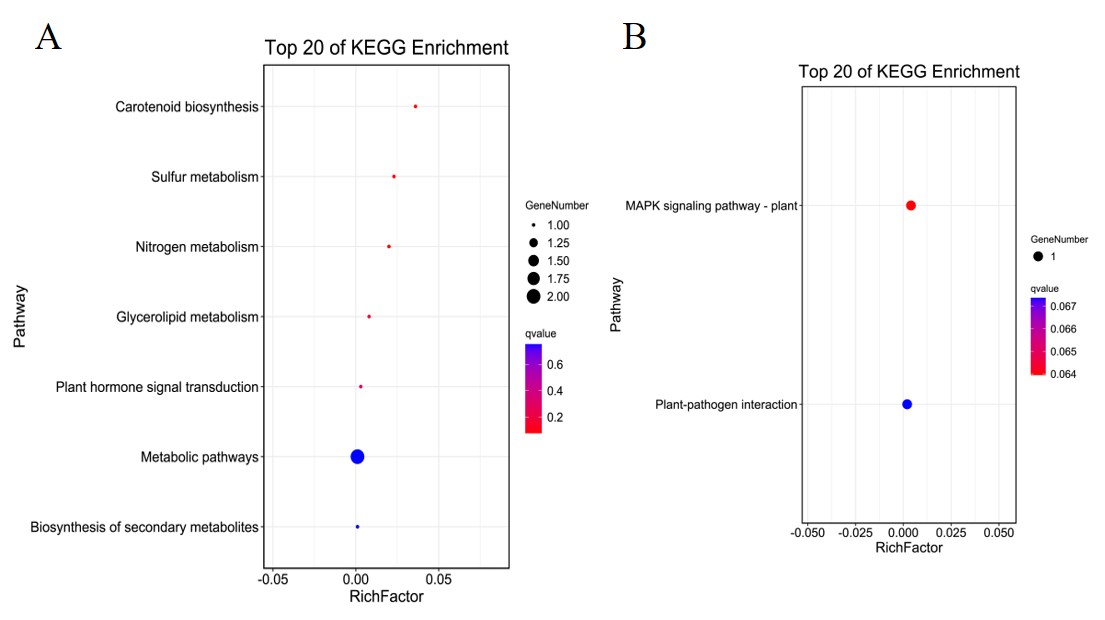

Supplement: Supplementary file 1 [file ijms-24-15762-s001.zip › Fig S9.jpg]
